# The Association of Work Overload with Burnout and Intent to Leave the Job Across the Healthcare Workforce During COVID-19

**DOI:** 10.1007/s11606-023-08153-z

**Published:** 2023-03-23

**Authors:** Lisa S. Rotenstein, Roger Brown, Christine Sinsky, Mark Linzer

**Affiliations:** 1 Brigham and Women’s Hospital and Harvard Medical School, Boston, MA USA; 2 University of Wisconsin School of Nursing, Madison, USA; 3 The American Medical Association, Chicago, IL USA; 4 Hennepin Healthcare and the University of Minnesota, Minneapolis, USA

## Abstract

**Background:**

Burnout has risen across healthcare workers during the pandemic, contributing to workforce turnover. While prior literature has largely focused on physicians and nurses, there is a need to better characterize and identify actionable predictors of burnout and work intentions across healthcare role types.

**Objective:**

To characterize the association of work overload with rates of burnout and intent to leave (ITL) the job in a large national sample of healthcare workers.

**Design:**

Cross-sectional survey study conducted between April and December 2020.

**Setting:**

A total of 206 large healthcare organizations.

**Participants:**

Physicians, nurses, other clinical staff, and non-clinical staff.

**Measures:**

Work overload, burnout, and ITL.

**Results:**

The sample of 43,026 respondents (mean response rate 44%) was comprised of 35.2% physicians, 25.7% nurses, 13.3% other clinical staff, and 25.8% non-clinical staff. The overall burnout rate was 49.9% (56.0% in nursing, 54.1% in other clinical staff, 47.3% in physicians, and 45.6% in non-clinical staff; *p* < 0.001 for difference). ITL was reported by 28.7% of healthcare workers, with nurses most likely to report ITL (41.0%), followed by non-clinical staff (32.6%), other clinical staff (32.1%), and physicians (24.3%) (*p* < 0.001 for difference). The prevalence of perceived work overload ranged from 37.1% among physicians to 47.4% in other clinical staff. In propensity-weighted models, work overload was significantly associated with burnout (adjusted risk ratio (ARR) 2.21 to 2.90) and intent to leave (ARR 1.73 to 2.10) across role types.

**Limitations:**

Organizations’ participation in the survey was voluntary.

**Conclusions:**

There are high rates of burnout and intent to leave the job across healthcare roles. Proactively addressing work overload across multiple role types may help with concerning trends across the healthcare workforce. This will require a more granular understanding of sources of work overload across different role types, and a commitment to matching work demands to capacity for all healthcare workers.

**Supplementary Information:**

The online version contains supplementary material available at 10.1007/s11606-023-08153-z.

## INTRODUCTION

Burnout, a phenomenon characterized by emotional exhaustion, depersonalization, and a lower sense of personal accomplishment, is of significant concern for the US healthcare system.^1^ In some studies, more than half of physicians report burnout,^2^ a condition associated with nearly twice the odds of intending to leave in some studies.^3^ Even before the pandemic, one-third of nurses reported burnout driving the decision to leave their jobs, with substantial human and financial consequences.^3,4^ Burnout is linked to decreased quality of care,^5^ and by contributing to turnover and reductions in clinical effort,^6^ has substantial costs for the healthcare system.^7,8^

While there was significant focus on burnout for physicians and nurses even prior to 2020, the COVID-19 pandemic has markedly increased stress among *all* types of healthcare workers,^9^ with evidence of the greatest prevalence of stress among nursing assistants, medical assistants, social workers, inpatient workers, women, and person of color.^9^ This stress was influenced by increasing work demands for all healthcare workers. Addressing the well-being of multiple role types is of crucial importance given widespread healthcare staffing shortages that impact quality and availability of healthcare, as well as role sustainability for those who remain in healthcare.^10^

We used data from a nationwide study of healthcare workers to answer two main questions related to healthcare worker experiences during the COVID-19 pandemic. First, how did the prevalence of burnout and intention to leave vary among physicians, nurses, other clinical staff, and non-clinical staff during the pandemic? Second, how do feelings of work overload vary across role types? Third, what is the association of work overload with burnout and intent to leave in different role types?

## METHODS

### Survey Design and Participants

This was an analysis of data from all respondents to the AMA Coping with COVID Survey, which was distributed to 206 organizations between April 2020 and March 2021. This analysis includes data from April to December 2020. Details of the Coping with COVID Study have previously been described.^11^ Briefly, the Coping with COVID Survey was administered by healthcare organizations across 30 states to healthcare personnel in clinical and non-clinical roles. Initially, 100 organizations were invited to participate. Many of these organizations had previously worked with the American Medical Association (AMA) on well-being initiatives.

Subsequently, other organizations heard of the survey through colleagues, emails, or news stories. Organizations with 100 physicians or more could register at a publicly available website at no cost. Institutions determined the frequency of reminder emails separately. Information was forwarded to a databank at Forward Health Group in Madison, Wisconsin. The study was deemed exempt from IRB review by the Hennepin Healthcare Institutional Review Board.^12^

### Survey Measures

The Coping with COVID Survey included questions about demographic characteristics of respondents, including occupation, self-reported race and ethnicity (including Asian/Pacific Islander, Black/African American, Hispanic/LatinX, Native American or American Indian, White, Other, or those who preferred not to answer (PNTA)), self-identified gender (female, male, non-binary/third gender, or PNTA), years in practice (1–5 years, 6–10 years, 11–15 years, 16–20 years, more than 20 years, or not reported), and practice setting (inpatient versus outpatient). Occupations were further categorized into residents and fellows, physicians, nurses, clinical staff, and non-clinical staff. For the purposes of this analysis, we only considered attending physicians in the physician category given the substantially different experiences of resident physicians in the workplace and their differential ability to modulate to or respond to their workload given training requirements. Clinical staff included pharmacists, nursing assistants, respiratory therapists, physical therapists, occupational therapists, speech therapists, medical assistants, and social workers. Non-clinical staff included housekeeping, administrative staff, receptionists, schedulers, lab or X-ray technicians, finance, food service, information technology support personnel, researchers without a clinical role, and laboratory staff.

Burnout was assessed using the Mini-Z single-item burnout measure. Validity of this single-item measure as compared to the emotional exhaustion subscale of the Maslach Burnout Inventory has been described previously.^13,14^ Respondents were additionally asked “What is the likelihood that you would leave your practice within two years?” This item was adapted from a previous national physician survey,^8^ and response options of “moderately,” “likely,” and “definitely” were considered positive for likely to leave their job. Across multiple studies, intention to leave has been demonstrated as a good predictor of actual turnover.^15^ Of note, this question was added to the survey on June 24, 2020, and thus was not administered to individuals in all organizations.

Finally, the survey queried participants regarding potential experiences related to burnout and work intentions. This included a measure of work overload, which was assessed by asking participants to rate their agreement with the following statement: “Due to the impact of COVID-19, I am experiencing work overload.” This question was assessed and scored on a 4-point scale (not at all, somewhat, moderately, or to a great extent), with the top two choices considered positive for the presence of work overload. See Appendix 1 for the full survey instrument.

### Statistical Analyses

We used descriptive statistics to describe distributions of gender, ethnicity, years in practice, practice setting, and state-level COVID load (COVID hospitalizations as a percent of total hospitalizations by state for each respondent’s state at the time point of response) among (1) physicians, (2) nurses, (3) non-physician and non-nurse clinical staff (hereafter referred to as “other clinical staff”), and (4) non-clinical staff. Advanced practice clinicians were not included in this analysis, although their findings have been included in prior Coping with COVID analyses.^12^ We then summarized proportions of respondents from each role type who met criteria for burnout and those who were classified as likely to leave their job within the next 2 years. Given missing responses to burnout and intent to leave questions, which served as the main outcomes in our study, we used descriptive statistics to characterize the gender, ethnicity, years in practice, and practice setting of respondents of each role type among those who completed the burnout and intent to leave questions versus the full sample. Finally, we summarized proportions of respondents from each role type who met criteria for work overload.

To minimize any covariate imbalance between respondents with work overload and those without, we used propensity score methodology to calculate the conditional probability (propensity) of respondents within each role group reporting work overload (versus not) given a set of covariates. We then used these conditional probabilities to propensity weight each role type sample that responded to the burnout and intent to leave questions using generalized boosted modeling.^17,18^

We subsequently built separate role type two-level (respondent within organization) random-intercept logistic regression models with standard errors clustered by organization determining the likelihood of burnout and intention to leave the job. Adjusted odds ratios, adjusted risk ratios, and adjusted risk differences were all estimated. ^16^

Given differential experiences with COVID burden and related stresses in the inpatient versus outpatient setting during the time that our survey was deployed, we sought to understand how the association between work overload and each of burnout and intent to leave varied by working in the inpatient versus outpatient setting. We thus used propensity score methodology to calculate the conditional probability (propensity) of respondents within each role group reporting work overload (versus not) and working in the inpatient setting (versus not) given a set of covariates.

We then used these conditional probabilities to propensity weight each role type sample that responded to the burnout and intent to leave questions using generalized boosted modeling.^17,18^ Finally, we built separate role type two-level random-intercept logistic regression models with standard errors clustered by organization that included an interaction term between work overload and practice setting, while adjusting for work overload and practice setting. Based on the marginal estimates from these models, we compared the percentage of respondents reporting burnout or intent to leave in the presence of work overload for the inpatient versus outpatient setting.

All analyses were conducted in Stata/SE version 17.0 (StataCorp, 2021). A threshold of *p* < 0.05 was used to denote statistical significance.

## RESULTS

### Sample Demographics

The sample consisted of a total of 43,026 individuals across 206 organizations (44% mean response rate). These organizations had a mean (SD) of 144.9 (117.9) physician respondents. A total of 117 (56.8%) were in the West, 18 (8.7%) in the South, 45 (21.8%) in the Northeast, and 26 (12.5%) in the Midwest.

The individuals in our sample included 15,142 physicians (35.2%), 11,040 nurses (25.7%), 5730 other clinical staff (13.3%), and 11,114 non-clinical staff (25.8%; see Table 1). Other clinical staff were comprised of 13.4% pharmacists (*n* = 768), 19.3% nursing assistants (*n* = 1106), 5.7% respiratory therapists (*n* = 329), 14.8% physical therapists (*n* = 847), 4.1% occupational therapists (*n* = 232), 2.5% speech therapists (*n* = 143), 21.4% medical assistants (*n* = 1225), and 18.9% social workers (*n* = 1080). Non-clinical staff were comprised of 2.1% housekeeping staff (*n* = 231), 47.5% administrative staff (*n* = 5284), 13.1% receptionist/scheduler staff (*n* = 1458), 7.5% lab or X-ray technicians (*n* = 831), 9.8% finance staff (*n* = 1084), 1.7% food service staff (*n* = 183), 7.1% information technology support staff (*n* = 785), 5.0% researchers without a clinical role (*n* = 560), and 6.3% laboratory staff (*n* = 698) (Table 1).

**Table 1:** Demographics of Study Sample by Role Type

	**Physicians** ***N* = 15,142** **(35.2%)**		**Nurses** ***N* = 11,040** **(25.7%)**		**Other clinical staff** ***N* = 5730** **(13.3%)**		**Non-clinical staff** ***N* = 11,114** **(25.8%)**	
	*N*	%	*N*	%	*N*	%	*N*	%
**Race and ethnicity**								
Asian/Pacific Islander	2371	15.7	700	6.9	328	5.5	527	4.7
Black/African American	289	1.9	773	7.6	523	9.1	1082	9.7
Hispanic/Latino	582	3.8	337	3.3	417	7.3	1034	9.3
Native American or American Indian	21	0.1	20	0.2	19	0.3	27	0.2
Prefer not to answer	1865	12.3	1358	13.4	690	12.0	1294	11.6
White	8314	54.9	6811	67.3	3372	58.9	6885	62.0
Other (please specify)	338	2.2	123	1.2	67	1.2	157	1.4
Missing	1362	9.0	918	8.3	314	5.5	108	1.0
**Self-reported gender**								
Female	6244	41.2	9348	84.7	4481	78.2	8140	73.2
Male	7697	50.8	742	6.7	801	14.0	2085	18.8
Non-binary/third gender	32	0.2	25	0.2	13	0.2	105	0.3
Prefer not to answer	1169	7.7	925	8.4	434	7.6	852	7.7
Missing	0	0.0	0	0.0	1	0.02	2	0.02
**Years in practice**								
1–5 years	2700	17.8	2257	20.5	1586	27.7	1916	17.2
6–10 years	2586	17.1	1925	17.4	1096	19.1	1166	10.5
11–15 years	2366	15.6	1527	13.8	800	14.0	1061	9.6
16–20 years	1962	13.0	1097	9.9	646	11.3	951	8.6
More than 20 years	5262	34.8	4032	36.5	1395	24.4	2531	22.8
Missing	266	1.8	202	1.8	207	3.6	3489	31.4
**Setting**								
Inpatient	7316	48.3	6831	61.9	2571	44.9	3748	33.7
Outpatient	5928	39.2	2351	21.3	2017	35.2	2423	21.8
Missing	1898	12.5	1858	16.8	1142	19.9	4943	44.5
**Specific roles**								
Physicians	15,142	100.0						
Nurses			11,040	100.0				
Pharmacist					768	13.4		
Nursing assistant					1106	19.3		
Respiratory therapist					329	5.7		
Physical therapist					847	14.8		
Occupational therapist					232	4.1		
Speech therapist					143	2.5		
Medical assistant					1225	21.4		
Social worker					1080	18.9		
Housekeeping							231	2.1
Administrative							5284	47.5
Receptionist/Scheduler							1458	13.1
Lab or X-ray technician							831	7.5
Finance							1084	9.8
Food service							183	1.7
IT support							785	7.1
Researcher (without clinical role)							560	5.0
Laboratory staff							698	6.3

More than two-thirds (*n* = 32,135; 66.2%) of all respondents identified as female (41.2% of physicians (*n* = 6244), 84.7% of nurses (*n* = 9348), 78.2% of other clinical staff (*n* = 4481), and 73.2% of non-clinical staff (*n* = 8140)). About half (*n* = 20,466; 47.77%) of respondents worked in the inpatient setting. This includes 48.3% (*n* = 7316) of physicians, 61.9% (*n* = 6831) of nurses, 44.9% (*n* = 2571) of other clinical staff, and 33.7% (*n* = 3748) of non-clinical staff. Over half of all role types identified as White individuals (*n* = 8314; 54.9% among physicians, *n* = 6811; 67.3% among nurses, *n* = 3372; 58.9% among other clinical staff, and *n* = 6885; 62.0% among non-clinical staff). Full demographic characteristics are displayed in Table 1.

### Burnout and Intent to Leave

A total of 40,301 individuals (93.6% of full sample) responded to the survey’s burnout question. Demographic characteristics of the individuals who responded to the survey’s burnout question in comparison to those of the full sample are displayed in Appendix 2. Many results were comparable, although there was a slight preponderance of inpatient workers and those preferring not to identify their race or ethnicity in those responding to the burnout question. Of the 40,301 respondents, 49.9% (*n* = 21,469) met the criteria for burnout. As shown in Table 2, nurses had the highest reported rates of burnout (56.0%, *n* = 5672), followed by other clinical staff (54.1%, *n* = 2928), physicians (47.3%, *n* = 6514), and non-clinical staff (45.6%, *n* = 5015). These values differed significantly across role types (*p* < 0.001).

**Table 2:** Proportions of Respondents Reporting Burnout, Intent to Leave, and Work Overload by Role Type

	**Burnout**	**Intent to leave**	**Work overload**
Physician	6514/13,780 (47.3%)	2280/9393 (24.3%)	5616/15,137 (37.1%)
Nurse	5672/10,122 (56.0%)	935/2280 (41.0%)	5164/11,011 (46.9%)
Clinical staff	2928/5415 (54.1%)	565/1759 (32.1%)	2715/5728 (47.4%)
Non-clinical staff	5015/11,005 (45.6%)	662/2033 (32.6%)	4941/11,103 (44.5%)

A total of 15,465 individuals (35.9% of full sample) responded to the intent to leave question. As expected, given that the intent to leave question was introduced later, those who responded to the ITL question had different distributions of some demographic characteristics (race/ethnicity, gender, years in practice, and practice setting) as compared to the full sample. Demographic characteristics of the individuals who responded to the survey’s intent to leave question in comparison to those of the full sample are displayed in Appendix 3. Of the 15,465 respondents, more than a quarter (28.7%; *n* = 15,465) endorsed an intent to leave their jobs. Nurses had the highest rates of reporting a high likelihood of intending to leave in the next 2 years (41.0%, *n* = 935), followed by other clinical staff (32.1%, *n* = 565), non-clinical staff (32.6%, *n* = 662), and physicians (24.3%, *n* = 2280) (Table 2). These rates differed significantly by role (*p* < 0.01).

### Perceptions of Work Overload

Non-physician and non-nurse clinical staff reported the highest prevalence of work overload at 47.4% (*n* = 2715). Nurses had a 46.9% (*n* = 5164) prevalence of work overload, followed by non-clinical staff at 44.5% (*n* = 4941) and physicians at 37.1% (*n* = 5616) (Table 2).

### Association of Work Overload with Burnout and Work Intentions

Propensity-weighted samples were well balanced in terms of demographic characteristics (defined as standard differences between “treatment” groups (work overload present versus work overload not present) of 0.2 or less) across the burnout respondents who met the criteria for work overload versus those who did not (Appendices 4a–4d). As shown in Table 3, based on the propensity-weighted sample, perceived work overload was significantly associated with burnout across role types. As our outcome was not rare, we present adjusted risk ratios to avoid overestimating the association between work overload and burnout. Adjusted odds ratios and adjusted risk differences are additionally presented in Table 3. Work overload was associated with a 2.42 (95% CI: 2.33, 2.50) times greater risk of burnout among physicians, 2.21 (95% CI: 2.12, 2.30) times greater risk of burnout among nurses, 2.29 (2.16, 2.43) greater risk of burnout among clinical staff, and 2.90 (95% CI: 2.77, 3.05) times greater risk of burnout among non-clinicians.

**Table 3:** Propensity-Weighted Associations of Work Overload* with Burnout by Role Type

	**ARR (95% CI)**	**ARD (95% CI)**	**AOR (95% CI)**
**Physicians** (*N* = 10,629; Organizations = 158) *McKelvey and Zavoina-Pseudo R*^*2*^ = 0.26	2.42 (2.33, 2.50)	0.43 (0.42, 0.45)	6.45 (5.90, 7.04)
**Nurses** (*N* = 6018; Organizations = 68) *McKelvey and Zavoina-Pseudo R*^*2*^ = 0.28	2.21 (2.12, 2.30)	0.43 (0.41, 0.45)	6.46 (5.97, 7.39)
**Clinical staff** (*N* = 3705; Organizations = 63) *McKelvey and Zavoina-Pseudo R*^*2*^ = 0.27	2.29 (2.16, 2.43)	0.43 (0.41, 0.46)	6.77 (6.09, 7.52)
**Non-clinical staff** (*N* = 4007; Organizations = 83) *McKelvey and Zavoina-Pseudo R*^*2*^ = 0.27	2.90 (2.77, 3.05)	0.47 (0.45, 0.49)	7.78 (7.20, 8.41)

*Work overload was defined as a response of “moderately” or “to a great extent” to the question “Due to the impact of COVID-19, I am experiencing work overload”

*ARR*, adjusted risk ratio; *ARD*, adjusted risk difference; *AOR*, adjusted odds ratio

Similarly, propensity-weighted samples were well balanced in terms of demographic characteristics across the intent to leave respondents who met the criteria for work overload versus those who did not (Appendices 5a–5d). As shown in Table 4, based on the propensity-weighted sample, work overload was associated with 1.73 (95% CI: 1.61, 1.87), 1.87 (95% CI: 1.65, 2.11), 2.04 (95% CI: 1.74, 2.38), and 2.10 (95% CI: 1.82, 2.43) times greater risk of intent to leave among physicians, nurses, clinical staff, and non-clinical staff, respectively. Adjusted odds ratios and adjusted risk differences for the associations between work overload and intent to leave by role type are additionally presented in Table 4.

**Table 4:** Propensity-Weighted Associations of Work Overload* with Intent to Leave by Role Type

	**ARR (95% CI)**	**ARD (95% CI)**	**AOR (95% CI)**
**Physicians** (*N* = 8817; Organizations = 119) *McKelvey and Zavoina-Pseudo R*^*2*^ = 0.15	1.73 (1.61, 1.87)	0.14 (0.12, 0.16)	2.19 (1.97, 2.44)
**Nurses** (*N* = 1979; Organizations = 41) *McKelvey and Zavoina-Pseudo R*^*2*^ = 0.12	1.87 (1.65, 2.11)	0.24 (0.20, 0.28)	2.72 (2.38, 3.10)
**Clinical staff** (*N* = 1451; Organizations = 40) *McKelvey and Zavoina-Pseudo R*^*2*^ = 0.11	2.04 (1.74, 2.38)	0.22 (0.17, 0.26)	2.83 (2.39, 3.34)
**Non-clinical staff** (*N* = 1071; Organizations = 51) *McKelvey and Zavoina-Pseudo R*^*2*^ = 0.15	2.10 (1.82, 2.43)	0.23 (0.19, 0.27)	2.95 (2.28, 3.80)

*Work overload was defined as a response of “moderately” or “to a great extent” to the question of: “Due to the impact of COVID-19, I am experiencing work overload”

*ARR*, adjusted risk ratio; *ARD*, adjusted risk difference; *AOR*, adjusted odds ratio

After propensity matching, covariates were well balanced between respondents with work overload versus those without and those working in the inpatient versus outpatient setting (data not shown). Multivariable models with an interaction term between work overload and practice setting revealed a significant interaction between work overload and inpatient versus outpatient practice setting when predicting the outcome of intent to leave for physicians (34.3% of inpatient physicians versus 31.1% of outpatient physicians experiencing work overload expressed an intent to leave; *p* = 0.045) (Table 5). Among nurses, 53.4% of nurses reporting work overload in the inpatient setting intended to leave their job in the next 2 years versus 45.2% of nurses in the outpatient setting (*p* = 0.01). Across other role types, there were no significant interactions between work overload and practice setting in predicting burnout or intent to leave the job (Table 5).

**Table 5:** Percent of Respondents Endorsing Burnout and Intent to Leave the Job, Comparing Respondents with Work Overload Practicing in the Inpatient versus Outpatient Setting

	**Burnout**				**Intent to leave**		
	**Inpatient with work overload** ***%***	**Outpatient with work overload** ***%***	***p*-value**		**Inpatient with work overload** ***%***	**Outpatient with work overload** ***%***	***p*-value**
**Physicians** (*N* = 10,629; Organizations = 158) *McKelvey and Zavoina-Pseudo R*^*2*^ = 0.259	74.4%	74.7%	0.88	**Physicians** (*N* = 8817; Organizations = 119) *McKelvey and Zavoina-Pseudo R*^*2*^ = 0.152	34.3%	31.1%	0.045
**Nurses** (*N* = 6018; Organizations = 68) *McKelvey and Zavoina-Pseudo R*^*2*^ = 0.283	78.9%	75.8%	0.09	**Nurses** (*N* = 1979; Organizations = 41) *McKelvey and Zavoina-Pseudo R*^*2*^ = 0.123	53.4%	45.2%	0.01
**Clinical staff** (*N* = 3705; Organizations = 63) *McKelvey and Zavoina-Pseudo R*^*2*^ = 0.266	75.3%	76.9%	0.43	**Clinical staff** (*N* = 1451; Organizations = 40) *McKelvey and Zavoina-Pseudo R*^*2*^ = 0.115	42.9%	42.1%	0.84
**Non-clinical staff** (*N* = 4007; Organizations = 83) *McKelvey and Zavoina-Pseudo R*^*2*^ = 0.274	72.4%	73.7%	0.53	**Non-clinical staff** (*N* = 1071; Organizations = 51) *McKelvey and Zavoina-Pseudo R*^*2*^ = 0.168	42.4%	45.1%	0.50

## DISCUSSION

In this cross-sectional, nationwide study of healthcare workers, we add to prior research focused mostly on physicians and nurses by demonstrating an elevated prevalence of burnout and intent to leave across healthcare role types, including non-physician and nurse clinical staff, and in non-clinical healthcare staff. We additionally identify work overload as being strongly associated with these outcomes across healthcare role types. Our findings are salient and urgent given staffing shortages across multiple healthcare role types ranging from home health aides to lab technicians, nursing assistants, and administrative staff, with significant implications of these shortages for the daily functioning of the healthcare system.^10^

Across all role types, work overload was a strong, independent predictor of burnout (up to 2.90 times greater risk of burnout with work overload) and intent to leave (up to 2.10 greater risk of intent to leave with work overload). While prior work has begun to examine the role of workload among physicians and nurses,^9,12,19–21^ our study expands this inquiry to other members of the healthcare workforce, including non-physician and non-nurse clinical staff such as nursing assistants and respiratory therapists, and non-clinical staff such as administrative and food service personnel.

Our data suggest that healthcare workers (especially nurses and other clinical staff) feel unable to meet what are at present unrealistic demands for productivity and efficiency, with downstream effects on well-being and work intentions. Approaches to workload reduction in medicine are haphazard, largely concentrated in trainees,^22–24^ and differ from other industries where exhausted workers (e.g., airline pilots) are not allowed to work and workload is closely monitored. Our findings suggest that a more standardized approach to measuring and limiting workload^25^ could contribute to reductions in burnout and turnover intentions. ^26,27^ Additionally, given evidence that enhanced job control moderates the relationship of workload and burnout,^28^ organizations may benefit from exploring interventions to modulate workload and ensure that employees have a sense of control over their work environment,^29^ even in times of unprecedented clinical volumes and ongoing pandemic stresses. Additional, systemic approaches should include ensuring sustainable compensation, adequate safeguards for mental and physical health, and readily accessible mental health resources for all members of the workforce. These goals will undoubtedly have to be achieved through both policy and local operational approaches. Indeed, substantive reforms to the healthcare sector may be required in order to create changes that enable us to appropriately value our caregivers, many of whom are women and persons of color.

The estimates of burnout presented in this study are similar or lower than those from other studies of burnout or its components during the height of the COVID-19 pandemic, with estimates varying by study location and the burnout instrument used.^30–32^ In general, across these studies, nurses and other clinical staff reported had a higher prevalence of burnout than physicians. For example, in a national survey of healthcare workers conducted before (2019) and twice during (2020 and 2021–2022) the COVID-19 pandemic, Sexton et al. described how burnout rates increased among some professions (nurses, clinical staff) from 2019 to 2020, and then notably increased further across role types by the time of the 2021–2022 survey.^30^ In a study by Guastello et al. between October and December 2020., nurses and technicians had higher burnout scores as compared to physicians.^33^ Similar trends of a higher rates of burnout non-physicians were reported in studies conducted in Japan^31^ and Italy^34^ during the COVID-19 pandemic. The consistency of the prevalence trends we have identified with those described in other studies underscores the need to enhance resources and attention to well-being for all members of healthcare workforce.

This study has numerous strengths. With over 40,000 respondents from 206 organizations, it represents the perceptions of diverse healthcare workers across inpatient and outpatient settings. It additionally queried respondents on multiple work-related experiences, allowing us to explore relationships between burnout, work intentions, and the relationship of workload to these outcomes. These strengths are balanced by limitations. First, organizations’ participation in the survey was voluntary, and distribution strategies and response rates varied, ultimately influencing our reporting of response rates at the organizational level, limiting our ability to report response rates by role type, and diminishing our ability to ensure that the distribution of the study sample acquired represented that of the overall US healthcare workforce. Additionally, limited information about organizational demographics precludes our assessing the extent to which organizations that responded to the survey represented all US healthcare organizations. Finally, this data was collected at the height of the COVID-19 pandemic; thus, the sentiments described by survey respondents may not be completely representative of their experiences at timepoints outside of a pandemic. Nevertheless, these data provide important insight into how work overload is associated with outcomes for both clinical and non-clinical workforce members, even in instances where the extent of work overload or healthcare circumstances may differ.

In conclusion, we demonstrate elevated levels of burnout and intent to leave across the healthcare workforce during COVID-19. Our findings uniquely provide evidence about the work intentions of *all* types of healthcare staff. They underscore how work overload is associated to these outcomes across role types and underscore the importance of measuring and modulating employees’ workload to facilitate the sustainability of healthcare delivery.

## Supplementary Information

Below is the link to the electronic supplementary material.

Supplementary file1 (DOCX 132 KB)
