# Supplementary material for: The Association of Work Overload with Burnout and Intent to Leave the Job Across the Healthcare Workforce During COVID-19
Source: J Gen Intern Med. 2023 Mar 23;38(8):1920–7. doi: 10.1007/s11606-023-08153-z (PMC10035977; doi:10.1007/s11606-023-08153-z)
Supplement: Supplementary file 1 — Supplementary file1 (DOCX 132 KB) [file 11606_2023_8153_MOESM1_ESM.docx]

**Appendix 1. Full Question List from AMA Coping with COVID Survey Instrument**

- 1. **1. The stress I experienced today is**
  2. a. Minimal
  3. b. Modest
  4. c. High
  5. d. Very high
  6. **2. I worry about exposing myself and my family to COVID**
  7. a. Not at all
  8. b. Somewhat
  9. c. Moderately
  10. d. To a great extent
  11. **3. Due to the impact of COVID 19, I am experiencing the following:**
  12. a. Anxiety or depression [not at all, somewhat, moderately, to a great extent]
  13. b. Work overload [not at all, somewhat, moderately, to a great extent]
  14. c. Concerns about childcare [not at all, somewhat, moderately, to a great extent]
  15. d. Worries that I have had to do things at work that compromise my integrity [not at all, somewhat, moderately, to a great extent]
  16. **4. How would the following improve your ability to sustain through the COVID crisis?**
  17. a. Staff or colleague support for inbox, documentation, and order entry [not at all, somewhat, moderately, to a great extent, N/A]
  18. b. Healthy food available at all hours [not at all, somewhat, moderately, to a great extent, N/A]
  19. c. Personal access to mental health care [not at all, somewhat, moderately, to a great extent, N/A]
  20. **5. Being part of the COVID-19 response has increased my sense of meaning and purpose**
  21. a. Not at all
  22. b. Somewhat
  23. c. Moderately
  24. d. To a great extent
  25. **6. I feel valued by my organization**
  26. a. Not at all
  27. b. Somewhat
  28. c. Moderately
  29. d. To a great extent

**7. What is the likelihood that you will reduce the number of hours you devote to clinical care over the next 12 months?**

a. None

- 1. b. Slight
  2. c. Moderate
  3. d. Likely
  4. e. Definitely
  5. **8. What is the likelihood that you would leave your practice within two years?**
  6. a. None
  7. b. Slight
  8. c. Moderate
  9. d. Likely
  10. e. Definitely

**9. Using your own definition of “burnout,” please choose one of the answers below:**

- 1. a. I enjoy my work. I have no symptoms of burnout
  2. b. I am under stress, and don’t always have as much energy as I did, but I don’t feel burned out.
  3. c. I am beginning to burn out and have one or more symptoms of burnout, e.g. emotional exhaustion
  4. d. The symptoms of burnout that I’m experiencing won’t go away. I think about work frustrations a lot.
  5. e. I feel completely burned out. I am at a point where I may need to seek help
  6. **10. Over the past two weeks, how often have you been bothered by little interest or pleasure in doing things?**
  7. a. Nearly every day
  8. b. More than half the days
  9. c. Several days
  10. d. Not at all

**11. Over the past two weeks, how often have you been bothered by feeling down, depressed, or hopeless?**

- 1. a. Nearly every day
  2. b. Nearly half the days
  3. c. Several days
  4. d. Not at all

**12. During the past 12 months have you had thoughts of taking your own life?**

- 1. a. Yes
  2. b. No
  3. **13.What may prevent you from seeking mental health services or support? (select all that apply)**
  4. a. I am concerned about what others would think if they knew I sought help
  5. b. I am concerned about confidentiality
  6. c. Such services are not accessible or convenient
  7. d. I am concerned this would impact my professional licensure
  8. e. I am concerned this would impact my employment (e.g. loss of hospital privileges)
  9. f. I cannot afford it
  10. g. I prefer other sources of help (family, friends, etc.)
  11. h. I prefer to handle my problems by myself
  12. i. None of the above
  13. **14.What else would you like to tell us about how your experience during the COVID 19 crisis?** (Note: Your anonymous answers may be viewed by your institution or practice manager) a. [[free text]]
  14. **15. Please specify your gender** a. Female
  15. b. Male
  16. c. Non-Binary/Third Gender
  17. d. Prefer not to answer
  18. **16. Please specify your ethnicity**
  19. a. White/Caucasian
  20. b. Hispanic/Latino
  21. c. Black/African American
  22. d. Native American or American Indian
  23. e. Asian/Pacific Islander
  24. f. Prefer not to answer
  25. g. Other (please specify)
  26. **17.Which of the following best describes you?**
  27. a. Administrative
  28. b. Advanced Practice Provider
  29. c. Finance
  30. d. Food service
  31. e. Housekeeping
  32. f. IT Support
  33. g. Lab or X-ray Technician
  34. h. Laboratory staff
  35. i. Medical Assistant
  36. j. Nurse
  37. k. Nursing Assistant
  38. l. Occupational Therapist
  39. m. Receptionist/Scheduler
  40. n. Resident or Fellow
  41. o. Pharmacist
  42. p. Physical Therapist
  43. q. Physician
  44. r. Researcher (without clinical role)
  45. s. Respiratory Therapist
  46. t. Social worker
  47. u. Speech Therapist
  48. v. Other (write-in)

**18. In what setting(s) do you currently spend your clinical/staff time? (“select all apply”)**

- 1. a. Hospital-based: ER or ICU
  2. b. Hospital-based: non-ER, non-ICU
  3. c. Ambulatory-based: COVID care
  4. d. Ambulatory-based: Non-COVID care
  5. e. Other (i.e. remote/telemedicine, etc.) (write-in)
  6. **19. How many years after training have you been in practice?**
  7. a. 1-5 years
  8. b. 6-10 years
  9. c. 11-15 years
  10. d. 16-20 years
  11. e. More than 20 years
  12. f. N/A

**20. Please indicate which of the following best describes your medical specialty.**

- 1. a. Allergy and Immunology
  2. b. Anesthesiology
  3. c. Cardiac/Thoracic Surgery
  4. d. Cardiovascular Diseases
  5. e. Critical Care Medicine
  6. f. Dentistry/Oral Surgery
  7. g. Dermatology
  8. h. Emergency Medicine
  9. i. Family Medicine
  10. j. Gastroenterology
  11. k. General Practice
  12. l. Hematology/Oncology
  13. m. Hospitalist
  14. n. Infectious Disease
  15. o. Internal Medicine – General, Primary Care
  16. p. Nephrology
  17. q. Neurological Surgery
  18. r. Neurology
  19. s. Obstetrics and Gynecology
  20. t. Oncology
  21. u. Ophthalmology
  22. v. Orthopedic Surgery
  23. w. Otolaryngology
  24. x. Palliative Care
  25. y. Pathology
  26. z. Pediatrics
  27. aa. Physical and Occupational Therapy
  28. bb. Physical and Rehabilitation Medicine
  29. cc. Plastic Surgery
  30. dd. Podiatry
  31. ee. Psychiatry
  32. ff. Pulmonary Disease
  33. gg. Radiation Oncology
  34. hh. Radiology
  35. ii. Rheumatology
  36. jj. Surgery, General
  37. kk. Urological Surgery
  38. ll. Vascular Surgery
  39. mm. Other, surgery-related specialty
  40. nn. Other, non-surgery-related specialty
  41. oo. N/A

| **Appendix 2. Characteristics of Population Who Responded to Burnout Question versus Full Sample** | | | | | | | | | | | | | | | | | | | | | | |
| --- | --- | --- | --- | --- | --- | --- | --- | --- | --- | --- | --- | --- | --- | --- | --- | --- | --- | --- | --- | --- | --- | --- |
|  | **Physicians** | | | | | **Nurses** | | | | | **Other Clinical Staff** | | | | | **Non-clinical Staff** | | | | | | |
|  | Full Sample  *N=15,142*  *(35.2%)* | | Burnout Question Respondents  *N = 13,780*  *(34.2%)* | | p-value | Full Sample  *N=11,040*  *(25.7%)* | | Burnout Question Respondents  *N = 10,118*  *(25.1%)* | | p-value | Full Sample  *N=5,730*  *(13.3%)* | | Burnout Question Respondents  *N = 5,412*  *(13.4%)* | | p-value | Full Sample  *N=11,114*  *(25.8%)* | | Burnout Question Respondents  *N = 10,991*  *(27.3%)* | | | p-value |  |
|  | N | % | N | % |  | N | % | N | % |  | N | % | N | % |  | N | % | N | % |  | |  |
| **Race and Ethnicity** |  |  |  |  |  |  |  |  |  |  |  |  |  |  |  |  |  |  |  |  | |  |
| Asian/Pacific Islander | 2371 | 15.7 | 2370 | 17.2 | <0.01 | 700 | 6.9 | 700 | 6.9 | <0.01 | 328 | 5.5 | 328 | 6.1 | <0.01 | 527 | 4.7 | 527 | 4.8 | <0.01 | |  |
| Black/African American | 289 | 1.9 | 289 | 2.1 |  | 773 | 7.6 | 773 | 7.6 |  | 523 | 9.1 | 523 | 9.7 |  | 1082 | 9.7 | 1082 | 9.8 |  |  |  |
| Hispanic/Latino | 582 | 3.8 | 582 | 4.2 |  | 337 | 3.3 | 337 | 3.3 |  | 417 | 7.3 | 417 | 7.7 |  | 1034 | 9.3 | 1034 | 9.4 |  |  |  |
| Native American or American Indian | 21 | 0.1 | 21 | 0.6% |  | 20 | 0.2 | 20 | 0.2 |  | 19 | 0.3 | 19 | 0.4 |  | 27 | 0.2 | 27 | 0.2 |  |  |  |
| Prefer not to answer | 1865 | 12.3 | 1865 | 13.5% |  | 1358 | 13.4 | 1357 | 19.9 |  | 690 | 12.0 | 690 | 12.7 |  | 1294 | 11.6 | 1293 | 11.8 |  |  |  |
| White | 8314 | 54.9 | 8314 | 60.3 |  | 6811 | 67.3 | 6807 | 67.3 |  | 3372 | 58.9 | 3366 | 62.2 |  | 6885 | 62.0 | 6870 | 62.5 |  |  |  |
| Other (please specify) | 338 | 2.2 | 338 | 2.5 |  | 123 | 1.2 | 123 | 1.2 |  | 67 | 1.2 | 67 | 1.2 |  | 157 | 1.4 | 157 | 1.4 |  |  |  |
| Missing | 1362 | 9.0 | 1 | 0.01 |  | 918 | 8.3 | 1 | 0.0 |  | 314 | 5.5 | 2 | 0.04 |  | 108 | 1.0 | 1 | 0.01 |  |  |  |
| **Self-Reported Gender** |  |  |  |  |  |  |  |  |  |  |  |  |  |  |  |  |  |  |  |  | |  |
| Female | 6244 | 41.2 | 5624 | 40.8 | 0.91 | 9348 | 84.7 | 8550 | 84.5 | 0.96 | 4481 | 78.2 | 4223 | 78.0 | 0.99 | 8140 | 73.2 | 8045 | 73.2 | <0.01 | |  |
| Male | 7697 | 50.8 | 7050 | 51.2 |  | 742 | 6.7 | 679 | 6.7 |  | 801 | 14.0 | 754 | 13.9 |  | 2085 | 18.8 | 2066 | 18.8 |  |  |  |
| Non-binary/third gender | 32 | 0.2 | 29 | 0.2 |  | 25 | 0.2 | 25 | 0.3 |  | 13 | 0.2 | 12 | 0.2 |  | 105 | 0.3 | 35 | 0.3 |  |  |  |
| Prefer not to answer | 1169 | 7.7 | 1077 | 7.8 |  | 925 | 8.4 | 864 | 8.5 |  | 434 | 7.6 | 422 | 7.8 |  | 852 | 7.7 | 844 | 7.7 |  |  |  |
| Missing | 0 | 0.0 | 0 | 0.0 |  | 0 | 0.0 | 0 | 0.0 |  | 1 | 0.02 | 1 | 0.0 |  | 2 | 0.02 | 1 | 0.0 |  |  |  |
| **Years in Practice** |  |  |  |  |  |  |  |  |  |  |  |  |  |  |  |  |  |  |  |  |  |  |
| 1-5 years | 2700 | 17.8 | 2364 | 17.2 | 0.58 | 2257 | 20.5 | 2077 | 20.5 | 0.99 | 1586 | 27.7 | 1504 | 27.8 | 0.99 | 1916 | 17.2 | 1887 | 17.2 | 0.99 | |  |
| 6-10 years | 2586 | 17.1 | 2342 | 17.0 |  | 1925 | 17.4 | 1752 | 17.3 |  | 1096 | 19.1 | 1037 | 19.2 |  | 1166 | 10.5 | 1159 | 10.5 |  |  |  |
| 11-15 years | 2366 | 15.6 | 2196 | 15.9 |  | 1527 | 13.8 | 1392 | 13.8 |  | 800 | 14.0 | 760 | 14.0 |  | 1061 | 9.6 | 1051 | 9.6 |  |  |  |
| 16-20 years | 1962 | 13.0 | 1802 | 13.1 |  | 1097 | 9.9 | 1001 | 9.9 |  | 646 | 11.3 | 621 | 11.5 |  | 951 | 8.6 | 938 | 8.5 |  |  |  |
| More than 20 years | 5262 | 34.8 | 4855 | 35.2 |  | 4032 | 36.5 | 3700 | 36.6 |  | 1395 | 24.4 | 1295 | 23.9 |  | 2531 | 22.8 | 2506 | 22.8 |  |  |  |
| Missing | 266 | 1.8 | 221 | 1.6 |  | 202 | 1.8 | 196 | 1.9 |  | 207 | 3.6 | 195 | 3.6 |  | 3489 | 31.4 | 3450 | 31.4 |  |  |  |
| **Setting** |  |  |  |  |  |  |  |  |  |  |  |  |  |  |  |  |  |  |  |  | |  |
| Inpatient | 7316 | 48.3 | 7316 | 53.1 | <0.01 | 6831 | 61.9 | 6829 | 67.5 | <0.01 | 2571 | 44.9 | 2569 | 47.5 | <0.01 | 3748 | 33.7 | 3741 | 34.0 | <0.01 | |  |
| Outpatient | 5928 | 39.2 | 5927 | 43.0 |  | 2351 | 21.3 | 2350 | 23.2 |  | 2017 | 35.2 | 2016 | 37.3 |  | 2423 | 21.8 | 2421 | 22.0 |  |  |  |
| Missing | 1898 | 12.5 | 537 | 3.9 |  | 1858 | 16.8 | 939 | 9.3 |  | 1142 | 19.9 | 827 | 15.3 |  | 4943 | 44.5 | 4829 | 43.9 |  |  |  |
| **Specific Roles** |  |  |  |  |  |  |  |  |  |  |  |  |  |  |  |  |  |  |  |  | |  |
| Pharmacist |  | | | | | | | | | | 768 | 13.4 | 711 | 13.1 | 0.62 |  | | | |  | |  |
| Nursing Assistant |  |  |  |  |  |  |  |  |  |  | 1106 | 19.3 | 995 | 18.4 |  |  |  |  |  |  |  |  |
| Respiratory Therapist |  |  |  |  |  |  |  |  |  |  | 329 | 5.7 | 285 | 5.3 |  |  |  |  |  |  |  |  |
| Physical Therapist |  |  |  |  |  |  |  |  |  |  | 847 | 14.8 | 799 | 14.8 |  |  |  |  |  |  |  |  |
| Occupational Therapist |  |  |  |  |  |  |  |  |  |  | 232 | 4.1 | 206 | 3.8 |  |  |  |  |  |  |  |  |
| Speech Therapist |  |  |  |  |  |  |  |  |  |  | 143 | 2.5 | 139 | 2.6 |  |  |  |  |  |  |  |  |
| Medical Assistant |  |  |  |  |  |  |  |  |  |  | 1225 | 21.4 | 1210 | 22.4% |  |  |  |  |  |  |  |  |
| Social Worker |  |  |  |  |  |  |  |  |  |  | 1080 | 18.9 | 1067 | 19.7% |  |  |  |  |  |  |  |  |
| Housekeeping |  |  |  |  |  |  |  |  |  |  |  | | | |  | 231 | 2.1 | 192 | 1.8 | 0.91 | |  |
| Administrative |  |  |  |  |  |  |  |  |  |  |  |  |  |  |  | 5284 | 47.5 | 5236 | 47.6 |  |  |  |
| Receptionist/Scheduler |  |  |  |  |  |  |  |  |  |  |  |  |  |  |  | 1458 | 13.1 | 1437 | 13.1 |  |  |  |
| Lab or X-Ray Technician |  |  |  |  |  |  |  |  |  |  |  |  |  |  |  | 831 | 7.5 | 825 | 7.5 |  |  |  |
| Finance |  |  |  |  |  |  |  |  |  |  |  |  |  |  |  | 1084 | 9.8 | 1080 | 9.8 |  |  |  |
| Food Service |  |  |  |  |  |  |  |  |  |  |  |  |  |  |  | 183 | 1.7 | 183 | 1.7 |  |  |  |
| IT Support |  |  |  |  |  |  |  |  |  |  |  |  |  |  |  | 785 | 7.1 | 783 | 7.1 |  |  |  |
| Researcher (without clinical role) |  |  |  |  |  |  |  |  |  |  |  |  |  |  |  | 560 | 5.0 | 558 | 5.1 |  |  |  |
| Laboratory Staff |  |  |  |  |  |  |  |  |  |  |  |  |  |  |  | 698 | 6.3 | 697 | 6.3 |  |  |  |

| **Appendix 3. Characteristics of Population Who Responded to Intent to Leave Question versus Full Sample** | | | | | | | | | | | | | | | | | | | | | | | |
| --- | --- | --- | --- | --- | --- | --- | --- | --- | --- | --- | --- | --- | --- | --- | --- | --- | --- | --- | --- | --- | --- | --- | --- |
|  | **Physicians** | | | | | **Nurses** | | | | | **Other Clinical Staff** | | | | | **Non-clinical Staff** | | | | | | | |
|  | Full Sample  *N=15,142*  *(35.2)* | | ITL Question Respondents  *N=9,393*  *(60.7%)* | | p-value | Full Sample  *N=11,040*  *(25.7%)* | | ITL Question Respondents  *N = 2,280*  *(14.7%)* | | p-value | Full Sample  *N=5,730*  *(13.3%)* | | ITL Question Respondents  *N = 1,759*  *(11.4%)* | | p-value | Full Sample  *N=11,114*  *(25.8%)* | | | ITL Question Respondents  *N = 2,033*  *(13.1)* | | | p-value |  |
|  | N | % | N | % |  | N | % | N | % |  | N | % | N | % |  | N | % | N | | % |  | |  |
| **Race and Ethnicity** |  |  |  |  |  |  |  |  |  |  |  |  |  |  |  |  |  |  | |  |  | |  |
| Asian/Pacific Islander | 2371 | 15.7 | 1821 | 19.4 | <0.01 | 700 | 6.9 | 42 | 1.8 | <0.01 | 328 | 5.5 | 81 | 4.6 | <0.01 | 527 | 4.7 | 55 | | 2.7 | <0.01 | |  |
| Black/African American | 289 | 1.9 | 143 | 1.5 |  | 773 | 7.6 | 55 | 2.4 |  | 523 | 9.1 | 55 | 3.1 |  | 1082 | 9.7 | 68 | | 3.3 |  |  |  |
| Hispanic/Latino | 582 | 3.8 | 382 | 4.1 |  | 337 | 3.3 | 30 | 1.3 |  | 417 | 7.3 | 49 | 2.8 |  | 1034 | 9.3 | 55 | | 2.7 |  |  |  |
| Native American or American Indian | 21 | 0.1 | 19 | 0.2 |  | 20 | 0.2 | 7 | 0.3 |  | 19 | 0.3 | 7 | 0.4 |  | 27 | 0.2 | 9 | | 0.4 |  |  |  |
| Prefer not to answer | 1865 | 12.3 | 1313 | 14.0 |  | 1358 | 13.4 | 285 | 12.5 |  | 690 | 12.0 | 168 | 9.6 |  | 1294 | 11.6 | 186 | | 9.2 |  |  |  |
| White | 8314 | 54.9 | 5489 | 58.4 |  | 6811 | 67.3 | 1837 | 80.6 |  | 3372 | 58.9 | 1379 | 78.4 |  | 6885 | 62.0 | 1640 | | 80.7 |  |  |  |
| Other (please specify) | 338 | 2.2 | 226 | 2.4 |  | 123 | 1.2 | 24 | 1.1 |  | 67 | 1.2 | 20 | 1.1 |  | 157 | 1.4 | 20 | | 1.0 |  |  |  |
| Missing | 1362 | 9.0 | 0 | 0.0 |  | 918 | 8.3 | 0 | 0.0 |  | 314 | 5.5 | 0 | 0.0 |  | 108 | 1.0 | 0 | | 0.0 |  |  |  |
| **Self-Reported Gender** |  |  |  |  |  |  |  |  |  |  |  |  |  |  |  |  |  |  | |  |  | |  |
| Female | 6244 | 41.2 | 3676 | 39.1 |  | 9348 | 84.7 | 1908 | 83.7 | 0.22 | 4481 | 78.2 | 1413 | 80.3 | <0.01 | 8140 | 73.2 | 1587 | | 78.1 | <0.01 | |  |
| Male | 7697 | 50.8 | 4910 | 52.3 | 0.008 | 742 | 6.7 | 147 | 6.5 |  | 801 | 14.0 | 214 | 12.2 |  | 2085 | 18.8 | 275 | | 13.5 |  |  |  |
| Non-binary/ third gender | 32 | 0.2 | 20 | 0.2 |  | 25 | 0.2 | 4 | 0.2 |  | 13 | 0.2 | 1 | 0.1 |  | 105 | 0.3 | 6 | | 0.3 |  |  |  |
| Prefer not to answer | 1169 | 7.7 | 787 | 8.4 |  | 925 | 8.4 | 221 | 9.7 |  | 434 | 7.6 | 131 | 7.5 |  | 852 | 7.7 | 165 | | 8.1 |  |  |  |
| Missing | 0 | 0.0 | 0 | 0.0 |  | 0 | 0.0 | 0 | 0.0 |  | 1 | 0.02 | 0 | 0 |  | 2 | 0.02 | 0 | | 0 |  |  |  |
| **Years in Practice** |  |  |  |  |  |  |  |  |  |  |  |  |  |  |  |  |  |  | |  |  |  |  |
| 1-5 years | 2700 | 17.8 | 1580 | 16.8 | <0.01 | 2257 | 20.5 | 530 | 23.2 | <0.01 | 1586 | 27.7 | 507 | 28.8 | 0.09 | 1916 | 17.2 | 518 | | 25.5 | <0.01 | |  |
| 6-10 years | 2586 | 17.1 | 1746 | 18.6 |  | 1925 | 17.4 | 443 | 19.4 |  | 1096 | 19.1 | 360 | 20.5 |  | 1166 | 10.5 | 275 | | 13.5 |  |  |  |
| 11-15 years | 2366 | 15.6 | 1653 | 17.6 |  | 1527 | 13.8 | 306 | 13.4 |  | 800 | 14.0 | 265 | 15.1 |  | 1061 | 9.6 | 212 | | 10.4 |  |  |  |
| 16-20 years | 1962 | 13.0 | 1289 | 13.7 |  | 1097 | 9.9 | 239 | 10.5 |  | 646 | 11.3 | 181 | 10.3 |  | 951 | 8.6 | 200 | | 9.8 |  |  |  |
| More than 20 years | 5262 | 34.8 | 3049 | 32.5 |  | 4032 | 36.5 | 734 | 32.2 |  | 1395 | 24.4 | 400 | 22.7 |  | 2531 | 22.8 | 470 | | 23.1 |  |  |  |
| Missing | 266 | 1.8 | 76 | 0.8 |  | 202 | 1.8 | 28 | 1.2 |  | 207 | 3.6 | 46 | 2.6 |  | 3489 | 31.4 | 358 | | 17.6 |  |  |  |
| **Setting** |  |  |  |  |  |  |  |  |  |  |  |  |  |  |  |  |  |  | |  |  | |  |
| Inpatient | 7316 | 48.3 | 4937 | 52.6 | <0.01 | 6831 | 61.9 | 1339 | 58.7 | <0.01 | 2571 | 44.9 | 665 | 37.8 | <0.01 | 3748 | 33.7 | 635 | | 31.2 | <0.01 | |  |
| Outpatient | 5928 | 39.2 | 4208 | 44.8 |  | 2351 | 21.3 | 708 | 31.1 |  | 2017 | 35.2 | 841 | 47.8 |  | 2423 | 21.8 | 599 | | 29.5 |  |  |  |
| Missing | 1898 | 12.5 | 248 | 2.6 |  | 1858 | 16.8 | 233 | 10.2 |  | 1142 | 19.9 | 253 | 14.4 |  | 4943 | 44.5 | 799 | | 39.3 |  |  |  |
| **Specific Roles** |  |  |  |  |  |  |  |  |  |  |  |  |  |  |  |  |  |  | |  |  | |  |
| Pharmacist |  | | | | | | | | | | 768 | 13.4 | 298 | 16.9 | <0.01 |  | | | | | | |  |
| Nursing Assistant |  |  |  |  |  |  |  |  |  |  | 1106 | 19.3 | 203 | 11.5 |  |  |  |  |  |  |  |  |  |
| Respiratory Therapist |  |  |  |  |  |  |  |  |  |  | 329 | 5.7 | 85 | 4.8 |  |  |  |  |  |  |  |  |  |
| Physical Therapist |  |  |  |  |  |  |  |  |  |  | 847 | 14.8 | 327 | 18.6 |  |  |  |  |  |  |  |  |  |
| Occupational Therapist |  |  |  |  |  |  |  |  |  |  | 232 | 4.1 | 73 | 4.2 |  |  |  |  |  |  |  |  |  |
| Speech Therapist |  |  |  |  |  |  |  |  |  |  | 143 | 2.5 | 28 | 1.6 |  |  |  |  |  |  |  |  |  |
| Medical Assistant |  |  |  |  |  |  |  |  |  |  | 1225 | 21.4 | 527 | 30.0 |  |  |  |  |  |  |  |  |  |
| Social Worker |  |  |  |  |  |  |  |  |  |  | 1080 | 18.9 | 218 | 12.4 |  |  |  |  |  |  |  |  |  |
| Housekeeping |  |  |  |  |  |  |  |  |  |  |  | | | | | 231 | 2.1 | 56 | | 1.8 | 0.91 | |  |
| Administrative |  |  |  |  |  |  |  |  |  |  |  |  |  |  |  | 5284 | 47.5 | 683 | | 47.6 |  |  |  |
| Receptionist/Scheduler |  |  |  |  |  |  |  |  |  |  |  |  |  |  |  | 1458 | 13.1 | 447 | | 13.1 |  |  |  |
| Lab or X-Ray Technician |  |  |  |  |  |  |  |  |  |  |  |  |  |  |  | 831 | 7.5 | 288 | | 7.5 |  |  |  |
| Finance |  |  |  |  |  |  |  |  |  |  |  |  |  |  |  | 1084 | 9.8 | 109 | | 9.8 |  |  |  |
| Food Service |  |  |  |  |  |  |  |  |  |  |  |  |  |  |  | 183 | 1.7 | 14 | | 1.7 |  |  |  |
| IT Support |  |  |  |  |  |  |  |  |  |  |  |  |  |  |  | 785 | 7.1 | 105 | | 7.1 |  |  |  |
| Researcher (without clinical role) |  |  |  |  |  |  |  |  |  |  |  |  |  |  |  | 560 | 5.0 | 89 | | 5.1 |  |  |  |
| Laboratory Staff |  |  |  |  |  |  |  |  |  |  |  |  |  |  |  | 698 | 6.3 | 242 | | 6.3 |  |  |  |

**Appendix 4. Covariate Balance for Unweighted versus Propensity-Weighted Samples of Respondents to Burnout Question, by Work Overload Status and Role Type**

1. **Physicians**

| **Unweighted** | | | | | | **Propensity-Weighted** | | | | |
| --- | --- | --- | --- | --- | --- | --- | --- | --- | --- | --- |
|  | **Work Overload Present** | | **Work Overload Not Present** | | **Standardized Difference** | **Work Overload Present** | | **Work Overload Not Present** | | **Standardized Difference** |
|  | **Mean** | **SD** | **Mean** | **SD** |  | **Mean** | **SD** | **Mean** | **SD** |  |
| **COVD Load Quartile** | | | | | | | | | | |
| COVID Quartile 1 | 27.9% | 44.8% | 23.8% | 42.6% | 0.093 | 25.5% | 43.6% | 25.3% | 43.5% | 0.005 |
| COVID Quartile 2 | 22.3% | 41.7% | 25.3% | 43.5% | -0.070 | 24.1% | 42.8% | 24.3% | 42.9% | -0.005 |
| COVID Quartile 3 | 9.3% | 29.0% | 8.8% | 28.3% | 0.019 | 9.1% | 28.8% | 8.9% | 28.5% | 0.006 |
| COVID Quartile 4 | 23.2% | 42.2% | 21.1% | 40.8% | 0.053 | 21.8% | 41.3% | 21.8% | 41.3% | 0.000 |
| COVID Quartile: Missing | 17.3% | 37.8% | 21.1% | 40.8% | -0.095 | 19.4% | 39.6% | 19.6% | 39.7% | -0.005 |
| **Race/Ethnicity** | | | | | | | | | | |
| Prefer Not to Answer | 18.5% | 38.8% | 10.5% | 30.6% | 0.234 | 13.6% | 34.2% | 13.5% | 34.1% | 0.003 |
| White/Caucasian | 55.3% | 49.7% | 63.5% | 48.2% | -0.167 | 60.2% | 48.9% | 60.3% | 48.9% | -0.003 |
| Hispanic/Latino | 4.7% | 21.2% | 3.9% | 19.4% | 0.038 | 4.2% | 20.2% | 4.2% | 20.1% | 0.001 |
| Black/African American | 2.1% | 14.4% | 2.1% | 14.3% | 0.004 | 2.1% | 14.3% | 2.1% | 14.3% | -0.001 |
| Native American or American Indian | 0.1% | 2.4% | 0.2% | 4.6% | -0.040 | 0.1% | 3.9% | 0.2% | 3.9% | -0.001 |
| Asian/Pacific Islander | 16.9% | 37.5% | 17.4% | 37.9% | -0.013 | 17.3% | 37.8% | 17.3% | 37.8% | 0.001 |
| Other (Please Specify) | 2.5% | 15.5% | 2.5% | 15.5% | 0.000 | 2.5% | 15.5% | 2.5% | 15.5% | 0.001 |
| Ethnicity Missing | 0.0% | 0.0% | 0.0% | 1.1% | -0.014 | 0.0% | 0.0% | 0.0% | 1.0% | -0.013 |
| **Gender** | | | | | | | | | | |
| Prefer not to answer | 11.4% | 31.8% | 5.6% | 23.0% | 0.215 | 7.8% | 26.8% | 7.8% | 26.7% | 0.001 |
| Male | 43.6% | 49.6% | 55.8% | 49.7% | -0.245 | 51.0% | 50.0% | 51.3% | 50.0% | -0.006 |
| Female | 44.7% | 49.7% | 38.4% | 48.6% | 0.127 | 41.0% | 49.2% | 40.8% | 49.1% | 0.005 |
| Non-binary/third gender | 0.4% | 6.0% | 0.1% | 3.4% | 0.053 | 0.2% | 4.7% | 0.2% | 4.4% | 0.007 |
| **Years in Practice** | | | | | | | | | | |
| 1-5 years in practice | 16.2% | 36.8% | 17.8% | 38.2% | -0.042 | 17.2% | 37.7% | 17.2% | 37.7% | 0.001 |
| 6-10 years in practice | 19.1% | 39.3% | 15.7% | 36.4% | 0.091 | 17.0% | 37.6% | 17.0% | 37.6% | 0 |
| 11-15 years in practice | 19.2% | 39.4% | 13.9% | 34.6% | 0.146 | 16.0% | 36.7% | 15.8% | 36.5% | 0.007 |
| 16-20 years in practice | 15.1% | 35.8% | 11.8% | 32.3% | 0.098 | 13.2% | 33.8% | 13.1% | 33.7% | 0.002 |
| 20+ years in practice | 28.6% | 45.2% | 39.3% | 48.9% | -0.225 | 35.0% | 47.7% | 35.3% | 47.8% | -0.006 |
| Year in practice missing | 1.8% | 13.2% | 1.5% | 12.2% | 0.021 | 1.6% | 12.4% | 1.6% | 12.5% | -0.003 |
| **Practice Setting** | | | | | | | | | | |
| Inpatient Setting | 52.5% | 49.9% | 53.5% | 49.9% | -0.02 | 53.3% | 49.9% | 53.2% | 49.9% | 0.002 |
| Outpatient Setting | 44.9% | 49.7% | 41.9% | 49.3% | 0.06 | 43.1% | 49.5% | 42.9% | 49.5% | 0.004 |
| Setting Missing | 2.6% | 16.0% | 4.7% | 21.1% | -0.11 | 3.6% | 18.6% | 3.9% | 19.4% | -0.015 |

1. **Nurses**

| **Unweighted** | | | | | | **Propensity-Weighted** | | | | |
| --- | --- | --- | --- | --- | --- | --- | --- | --- | --- | --- |
|  | **Work Overload Present** | | **Work Overload Not Present** | | **Standardized Difference** | **Work Overload Group** | | **Work Overload Not Present** | | **Standardized Difference** |
|  | **Mean** | **SD** | **Mean** | **SD** |  | **Mean** | **SD** | **Mean** | **SD** |  |
| **COVD Load** |  |  |  |  |  |  |  |  |  |  |
| COVID Load Present | 10.6% | 30.8% | 13.2% | 33.8% | -0.079 | 10.8% | 31.1% | 12.9% | 33.5% | -0.063 |
| COVID Load Missing | 89.4% | 30.8% | 86.8% | 33.8% | 0.079 | 89.2% | 31.1% | 87.1% | 33.5% | 0.063 |
| **Race/Ethnicity** |  |  |  |  |  |  |  |  |  |  |
| Prefer Not to Answer | 17.1% | 37.7% | 10.0% | 29.9% | 0.21 | 13.4% | 34.1% | 13.3% | 34.0% | 0.002 |
| White/Caucasian | 63.3% | 48.2% | 71.0% | 45.4% | -0.163 | 67.3% | 46.9% | 67.4% | 46.9% | -0.001 |
| Hispanic/Latino | 3.8% | 19.1% | 2.9% | 16.8% | 0.047 | 3.3% | 17.9% | 3.3% | 17.9% | 0 |
| Black/African American | 6.7% | 25.0% | 8.5% | 27.9% | -0.067 | 7.6% | 26.5% | 7.6% | 26.5% | -0.001 |
| Native American or American Indian | 0.2% | 4.7% | 0.2% | 4.1% | 0.012 | 0.2% | 4.4% | 0.2% | 4.4% | 0 |
| Asian/Pacific Islander | 7.5% | 26.4% | 6.3% | 24.4% | 0.048 | 6.9% | 25.4% | 6.9% | 25.4% | 0 |
| Other (Please Specify) | 1.3% | 11.3% | 1.1% | 10.6% | 0.013 | 1.2% | 10.9% | 1.2% | 10.9% | 0 |
| Ethnicity Missing | 0.0% | 1.4% | 0.0% | 0.0% | 0.021 | 0.0% | 1.4% | 0.0% | 0.0% | 0.02 |
| **Gender** |  |  |  |  |  |  |  |  |  |  |
| Prefer not to answer | 11.6% | 32.1% | 5.7% | 23.1% | 0.213 | 8.6% | 28.1% | 8.3% | 27.6% | 0.012 |
| Male | 6.4% | 24.4% | 7.0% | 25.6% | -0.026 | 6.6% | 24.8% | 6.9% | 25.3% | -0.01 |
| Female | 81.7% | 38.7% | 87.1% | 33.5% | -0.151 | 84.5% | 36.2% | 84.6% | 36.1% | -0.003 |
| Non-binary/third gender | 0.3% | 5.7% | 0.2% | 4.1% | 0.031 | 0.3% | 5.0% | 0.2% | 4.7% | 0.006 |
| **Years in Practice** |  |  |  |  |  |  |  |  |  |  |
| 1-5 years in practice | 24.1% | 42.8% | 17.2% | 37.7% | 0.173 | 0.207 | 0.405 | 0.203 | 0.403 | 0.008 |
| 6-10 years in practice | 18.8% | 39.0% | 16.0% | 36.6% | 0.074 | 0.174 | 0.379 | 0.172 | 0.377 | 0.005 |
| 11-15 years in practice | 14.2% | 34.9% | 13.3% | 34.0% | 0.026 | 0.137 | 0.344 | 0.139 | 0.345 | -0.003 |
| 16-20 years in practice | 9.4% | 29.1% | 10.4% | 30.5% | -0.034 | 0.099 | 0.298 | 0.098 | 0.298 | 0.002 |
| 20+ years in practice | 31.2% | 46.3% | 41.6% | 49.3% | -0.215 | 0.363 | 0.481 | 0.369 | 0.483 | -0.011 |
| Year in practice missing | 2.3% | 15.0% | 1.6% | 12.6% | 0.05 | 0.020 | 0.139 | 0.019 | 0.137 | 0.004 |
| **Practice Setting** |  |  |  |  |  |  |  |  |  |  |
| Inpatient Setting | 69.5% | 46.1% | 65.7% | 47.5% | 0.081 | 67.4% | 46.9% | 67.3% | 46.9% | 0.004 |
| Outpatient Setting | 22.0% | 41.5% | 24.3% | 42.9% | -0.054 | 23.3% | 42.3% | 23.4% | 42.3% | -0.001 |
| Setting Missing | 8.5% | 27.9% | 10.0% | 30.0% | -0.051 | 9.2% | 28.9% | 9.4% | 29.2% | -0.006 |

1. **Other Clinical Staff**

| **Unweighted** | | | | | | **Propensity-Weighted** | | | | |
| --- | --- | --- | --- | --- | --- | --- | --- | --- | --- | --- |
|  | **Work Overload Present** | | **Work Overload Not Present** | | **Standardized Difference** | **Work Overload Present** | | **Work Overload Not Present** | | **Standardized Difference** |
|  | **Mean** | **SD** | **Mean** | **SD** |  | **Mean** | **SD** | **Mean** | **SD** |  |
| **COVD Load Quartile** | | | | | | | | | | |
| COVID Quartile 1 | 21.7% | 41.2% | 24.2% | 42.8% | -0.059 | 22.8% | 42.0% | 23.0% | 42.1% | -0.004 |
| COVID Quartile 2 | 9.3% | 29.1% | 8.0% | 27.1% | 0.047 | 8.7% | 28.2% | 8.6% | 28.1% | 0.003 |
| COVID Quartile 3 | 33.7% | 47.3% | 30.8% | 46.2% | 0.062 | 32.3% | 46.8% | 32.1% | 46.7% | 0.004 |
| COVID Quartile 4 | 20.3% | 40.2% | 18.5% | 38.8% | 0.047 | 19.3% | 39.5% | 19.3% | 39.4% | 0.002 |
| COVID Quartile: Missing | 14.9% | 35.7% | 18.6% | 38.9% | -0.097 | 16.8% | 37.4% | 16.9% | 37.5% | -0.004 |
| **Race/Ethnicity** | | | | | | | | | | |
| Prefer Not to Answer | 15.0% | 35.7% | 10.6% | 30.8% | 0.133 | 12.8% | 33.4% | 12.7% | 33.3% | 0.001 |
| White/Caucasian | 59.4% | 49.1% | 64.9% | 47.7% | -0.113 | 62.3% | 48.5% | 62.3% | 48.5% | 0 |
| Hispanic/Latino | 8.9% | 28.5% | 6.6% | 24.8% | 0.087 | 7.7% | 26.6% | 7.7% | 26.6% | 0.001 |
| Black/African American | 9.0% | 28.7% | 10.3% | 30.3% | -0.041 | 9.7% | 29.6% | 9.6% | 29.5% | 0.001 |
| Native American or American Indian | 0.3% | 5.8% | 0.4% | 6.0% | -0.003 | 0.3% | 5.7% | 0.3% | 5.7% | -0.001 |
| Asian/Pacific Islander | 5.9% | 23.6% | 6.2% | 24.1% | -0.013 | 6.0% | 23.8% | 6.0% | 23.8% | 0 |
| Other (Please Specify) | 1.4% | 11.7% | 1.1% | 10.4% | 0.029 | 1.2% | 11.0% | 1.2% | 11.0% | 0.001 |
| Ethnicity Missing | 0.0% | 0.0% | 0.1% | 2.7% | -0.038 | 0.0% | 0.0% | 0.1% | 2.6% | -0.036 |
| **Gender** | | | | | | | | | | |
| Prefer not to answer | 9.7% | 29.6% | 6.0% | 23.7% | 0.138 | 7.8% | 26.8% | 7.6% | 26.5% | 0.008 |
| Male | 12.0% | 32.5% | 15.7% | 36.4% | -0.107 | 13.8% | 34.5% | 14.0% | 34.7% | -0.005 |
| Female | 77.9% | 41.5% | 78.1% | 41.3% | -0.004 | 78.1% | 41.3% | 78.2% | 41.3% | -0.001 |
| Non-binary/third gender | 0.3% | 5.8% | 0.1% | 3.3% | 0.049 | 0.2% | 4.7% | 0.2% | 4.1% | 0.011 |
| **Years in Practice** | | | | | | | | | | |
| 1-5 years in practice | 29.8% | 45.7% | 25.9% | 43.8% | 0.088 | 27.9% | 44.8% | 27.7% | 44.7% | 0.004 |
| 6-10 years in practice | 20.9% | 40.6% | 17.5% | 38.0% | 0.086 | 19.2% | 39.4% | 19.2% | 39.4% | 0.001 |
| 11-15 years in practice | 15.0% | 35.7% | 13.1% | 33.8% | 0.055 | 14.0% | 34.7% | 14.0% | 34.7% | 0.001 |
| 16-20 years in practice | 9.9% | 29.9% | 13.0% | 33.6% | -0.096 | 11.4% | 31.8% | 11.6% | 32.0% | -0.006 |
| 20+ years in practice | 21.0% | 40.8% | 26.7% | 44.2% | -0.132 | 23.9% | 42.7% | 24.0% | 42.7% | -0.001 |
| Year in practice missing | 3.3% | 17.9% | 3.9% | 19.3% | -0.029 | 3.6% | 18.6% | 3.6% | 18.7% | -0.003 |
| **Practice Setting** | | | | | | | | | | |
| Inpatient Setting | 47.4% | 49.9% | 47.6% | 49.9% | -0.004 | 47.3% | 49.9% | 47.3% | 49.9% | 0 |
| Outpatient Setting | 38.2% | 48.6% | 36.4% | 48.1% | 0.037 | 37.3% | 48.4% | 37.3% | 48.4% | 0 |
| Setting Missing | 14.5% | 35.2% | 16.1% | 36.7% | -0.045 | 15.4% | 36.1% | 15.4% | 36.1% | 0 |

1. **Non-Clinical Staff**

| **Unweighted** | | | | | | **Propensity-Weighted** | | | | |
| --- | --- | --- | --- | --- | --- | --- | --- | --- | --- | --- |
|  | **Work Overload Present** | | **Work Overload Not Present** | | **Standardized Difference** | **Work Overload Group** | | **Work Overload Not Present** | | **Standardized Difference** |
|  | **Mean** | **SD** | **Mean** | **SD** |  | **Mean** | **SD** | **Mean** | **SD** |  |
| **COVD Load** |  |  |  |  |  |  |  |  |  |  |
| COVID Load Present | 15.9% | 36.6% | 16.6% | 37.2% | -0.017 | 15.7% | 36.4% | 16.6% | 37.2% | -0.026 |
| COVID Load Missing | 84.1% | 36.6% | 83.4% | 37.2% | 0.017 | 84.3% | 36.4% | 83.4% | 37.2% | 0.026 |
| **Race/Ethnicity** |  |  |  |  |  |  |  |  |  |  |
| Prefer Not to Answer | 14.3% | 35.0% | 9.7% | 29.6% | 0.143 | 12.0% | 32.5% | 11.5% | 31.9% | 0.016 |
| White/Caucasian | 60.7% | 48.8% | 63.9% | 48.0% | -0.066 | 62.4% | 48.5% | 62.6% | 48.4% | -0.005 |
| Hispanic/Latino | 9.8% | 29.8% | 9.1% | 28.7% | 0.027 | 9.5% | 29.3% | 9.4% | 29.2% | 0.004 |
| Black/African American | 8.3% | 27.6% | 11.1% | 31.4% | -0.094 | 9.6% | 29.5% | 10.1% | 30.1% | -0.015 |
| Native American or American Indian | 0.2% | 4.7% | 0.3% | 5.1% | -0.008 | 0.2% | 4.9% | 0.2% | 5.0% | -0.001 |
| Asian/Pacific Islander | 5.1% | 21.9% | 4.6% | 20.9% | 0.023 | 4.8% | 21.5% | 4.8% | 21.3% | 0.003 |
| Other (Please Specify) | 1.5% | 12.3% | 1.3% | 11.5% | 0.016 | 1.4% | 11.7% | 1.4% | 11.7% | 0.001 |
| Ethnicity Missing | 0.0% | 1.4% | 0.0% | 0.0% | 0.021 | 0.0% | 1.4% | 0.0% | 0.0% | 0.022 |
| **Gender** |  |  |  |  |  |  |  |  |  |  |
| Prefer not to answer | 10.1% | 30.2% | 5.7% | 23.2% | 0.167 | 7.8% | 26.9% | 7.4% | 26.2% | 0.016 |
| Male | 17.5% | 38.0% | 19.8% | 39.9% | -0.058 | 18.5% | 38.8% | 19.0% | 39.3% | -0.014 |
| Female | 71.9% | 45.0% | 74.3% | 43.7% | -0.054 | 73.3% | 44.2% | 73.2% | 44.3% | 0.001 |
| Non-binary/third gender | 0.4% | 6.5% | 0.2% | 4.8% | 0.035 | 0.3% | 5.7% | 0.3% | 5.5% | 0.004 |
| Missing | 0.0% | 1.4% | 0.0% | 0.0% | 0.021 | 0.0% | 1.4% | 0.0% | 0.0% | 0.02 |
| **Years in Practice** |  |  |  |  |  |  |  |  |  |  |
| 1-5 years in practice | 17.8% | 38.3% | 16.6% | 37.2% | 0.031 | 17.3% | 37.8% | 17.1% | 37.6% | 0.006 |
| 6-10 years in practice | 12.2% | 32.8% | 9.2% | 28.9% | 0.1 | 10.9% | 31.1% | 10.2% | 30.3% | 0.022 |
| 11-15 years in practice | 10.6% | 30.7% | 8.8% | 28.3% | 0.061 | 9.7% | 29.6% | 9.5% | 29.3% | 0.007 |
| 16-20 years in practice | 9.2% | 28.9% | 8.0% | 27.1% | 0.042 | 8.7% | 28.1% | 8.4% | 27.8% | 0.009 |
| 20+ years in practice | 22.6% | 41.8% | 22.9% | 42.0% | -0.008 | 22.6% | 41.8% | 23.0% | 42.1% | -0.011 |
| Year in practice missing | 27.6% | 44.7% | 34.5% | 47.5% | -0.148 | 30.9% | 46.2% | 31.8% | 46.6% | -0.019 |
| **Practice Setting** |  |  |  |  |  |  |  |  |  |  |
| Inpatient Setting | 34.4% | 47.5% | 33.7% | 47.3% | 0.014 | 33.9% | 47.3% | 34.2% | 47.4% | -0.005 |
| Outpatient Setting | 24.4% | 42.9% | 20.2% | 40.1% | 0.101 | 22.6% | 41.8% | 21.5% | 41.1% | 0.026 |
| Setting Missing | 41.3% | 49.2% | 46.1% | 49.8% | -0.098 | 43.5% | 49.6% | 44.3% | 49.7% | -0.017 |

**Appendix 5. Covariate Balance for Unweighted versus Propensity-Weighted Samples of Respondents to Intent to Leave Question, by Work Overload Status and Role Type**

1. **Physicians**

| **Unweighted** | | | | | | **Propensity-Weighted** | | | | |
| --- | --- | --- | --- | --- | --- | --- | --- | --- | --- | --- |
|  | **Work Overload Present** | | **Work Overload Not Present** | | **Standardized Difference** | **Work Overload Present** | | **Work Overload Not Present** | | **Standardized Difference** |
|  | **Mean** | **SD** | **Mean** | **SD** |  | **Mean** | **SD** | **Mean** | **SD** |  |
| **COVD Load Quartile** | | | | | | | | | | |
| COVID Quartile 1 | 33.0% | 47.0% | 29.0% | 45.4% | 0.087 | 30.6% | 46.1% | 30.5% | 46.0% | 0.003 |
| COVID Quartile 2 | 27.7% | 44.8% | 33.9% | 47.3% | -0.133 | 31.5% | 46.4% | 31.5% | 46.5% | -0.001 |
| COVID Quartile 3 | 6.0% | 23.7% | 5.3% | 22.3% | 0.031 | 5.5% | 22.9% | 5.5% | 22.9% | 0 |
| COVID Quartile 4 | 30.5% | 46.1% | 29.0% | 45.4% | 0.033 | 29.6% | 45.6% | 29.7% | 45.7% | -0.002 |
| COVID Quartile: Missing | 2.8% | 16.6% | 2.9% | 16.7% | -0.002 | 2.8% | 16.5% | 2.8% | 16.5% | 0 |
| **Race/Ethnicity** | | | | | | | | | | |
| Prefer Not to Answer | 19.1% | 39.3% | 10.6% | 30.8% | 0.247 | 13.9% | 34.6% | 13.8% | 34.5% | 0.005 |
| White/Caucasian | 53.4% | 49.9% | 61.8% | 48.6% | -0.171 | 58.4% | 49.3% | 58.5% | 49.3% | -0.002 |
| Hispanic/Latino | 4.5% | 20.6% | 3.8% | 19.1% | 0.033 | 4.1% | 19.8% | 4.1% | 19.8% | 0.001 |
| Black/African American | 1.6% | 12.6% | 1.5% | 12.0% | 0.012 | 1.5% | 12.1% | 1.5% | 12.1% | 0 |
| Native American or American Indian | 0.1% | 2.8% | 0.3% | 5.3% | -0.045 | 0.2% | 4.1% | 0.2% | 4.5% | -0.009 |
| Asian/Pacific Islander | 19.1% | 39.3% | 19.6% | 39.7% | -0.013 | 19.5% | 39.6% | 19.5% | 39.6% | 0 |
| Other (Please Specify) | 2.3% | 14.9% | 2.5% | 15.6% | -0.013 | 2.4% | 15.2% | 2.4% | 15.4% | -0.004 |
| **Gender** | | | | | | | | | | |
| Prefer not to answer | 12.0% | 32.5% | 6.0% | 23.7% | 0.218 | 8.4% | 27.7% | 8.2% | 27.5% | 0.005 |
| Male | 44.6% | 49.7% | 57.3% | 49.5% | -0.254 | 52.4% | 49.9% | 52.4% | 49.9% | -0.001 |
| Female | 43.1% | 49.5% | 36.5% | 48.2% | 0.134 | 39.1% | 48.8% | 39.1% | 48.8% | -0.001 |
| Non-binary/third gender | 0.3% | 5.4% | 0.2% | 4.0% | 0.03 | 0.2% | 4.4% | 0.2% | 4.3% | 0.002 |
| **Years in Practice** | | | | | | | | | | |
| 1-5 years in practice | 14.9% | 35.6% | 18.1% | 38.5% | -0.084 | 16.7% | 37.3% | 16.8% | 37.4% | -0.003 |
| 6-10 years in practice | 20.2% | 40.2% | 17.5% | 38.0% | 0.07 | 18.6% | 38.9% | 18.6% | 38.9% | -0.001 |
| 11-15 years in practice | 20.6% | 40.5% | 15.6% | 36.3% | 0.132 | 17.6% | 38.1% | 17.6% | 38.0% | 0 |
| 16-20 years in practice | 15.7% | 36.4% | 12.4% | 33.0% | 0.097 | 13.8% | 34.5% | 13.8% | 34.5% | 0.001 |
| 20+ years in practice | 27.3% | 44.5% | 35.9% | 48.0% | -0.183 | 32.6% | 46.9% | 32.5% | 46.8% | 0.002 |
| Year in practice missing | 1.2% | 10.9% | 0.5% | 7.4% | 0.074 | 0.8% | 8.9% | 0.8% | 8.7% | 0.003 |
| **Practice Setting** | | | | | | | | | | |
| Inpatient Setting | 50.8% | 50.0% | 53.7% | 49.9% | -0.059 | 52.6% | 49.9% | 52.7% | 49.9% | -0.001 |
| Outpatient Setting | 47.3% | 49.9% | 43.1% | 49.5% | 0.084 | 44.7% | 49.7% | 44.7% | 49.7% | 0.001 |
| Setting Missing | 1.9% | 13.7% | 3.1% | 17.4% | -0.076 | 2.6% | 16.0% | 2.6% | 16.0% | 0 |

1. **Nurses**

| **Unweighted** | | | | | | **Propensity-Weighted** | | | | |
| --- | --- | --- | --- | --- | --- | --- | --- | --- | --- | --- |
|  | **Work Overload Present** | | **Work Overload Not Present** | | **Standardized Difference** | **Work Overload Present** | | **Work Overload Not Present** | | **Standardized Difference** |
|  | **Mean** | **SD** | **Mean** | **SD** |  | **Mean** | **SD** | **Mean** | **SD** |  |
| **COVD Load Quartile** | | | | | | | | | | |
| COVID Quartile 1 | 15.9% | 36.5% | 25.4% | 43.5% | -0.239 | 20.0% | 40.0% | 20.4% | 40.3% | -0.011 |
| COVID Quartile 2 | 2.2% | 14.6% | 2.5% | 15.7% | -0.024 | 2.4% | 15.2% | 2.4% | 15.2% | 0 |
| COVID Quartile 3 | 42.2% | 49.4% | 43.7% | 49.6% | -0.029 | 42.9% | 49.5% | 43.5% | 49.6% | -0.013 |
| COVID Quartile 4 | 37.7% | 48.5% | 26.5% | 44.2% | 0.237 | 32.9% | 47.0% | 32.1% | 46.7% | 0.019 |
| COVID Quartile: Missing | 2.1% | 14.3% | 1.8% | 13.4% | 0.019 | 1.9% | 13.6% | 1.7% | 13.0% | 0.014 |
| **Race/Ethnicity** | | | | | | | | | | |
| Prefer Not to Answer | 15.7% | 36.4% | 8.3% | 27.6% | 0.224 | 12.7% | 33.3% | 12.1% | 32.6% | 0.019 |
| White/Caucasian | 77.3% | 41.9% | 84.8% | 35.9% | -0.189 | 80.6% | 39.6% | 81.0% | 39.2% | -0.011 |
| Hispanic/Latino | 1.3% | 11.4% | 1.3% | 11.4% | 0 | 1.3% | 11.3% | 1.5% | 12.1% | -0.016 |
| Black/African American | 2.2% | 14.6% | 2.7% | 16.3% | -0.037 | 2.2% | 14.8% | 2.2% | 14.7% | 0.002 |
| Native American or American Indian | 0.3% | 5.6% | 0.3% | 5.5% | 0.001 | 0.3% | 5.5% | 0.3% | 5.5% | -0.001 |
| Asian/Pacific Islander | 1.9% | 13.8% | 1.7% | 13.0% | 0.016 | 1.8% | 13.4% | 1.9% | 13.5% | -0.001 |
| Other (Please Specify) | 1.2% | 11.1% | 0.8% | 9.0% | 0.042 | 1.1% | 10.3% | 1.1% | 10.3% | 0.001 |
| **Gender** | | | | | | | | | | |
| Prefer not to answer | 11.7% | 32.1% | 7.1% | 25.7% | 0.155 | 9.8% | 29.7% | 10.0% | 29.9% | -0.006 |
| Male | 6.0% | 23.7% | 7.1% | 25.7% | -0.046 | 6.6% | 24.8% | 6.8% | 25.1% | -0.008 |
| Female | 82.3% | 38.2% | 85.5% | 35.2% | -0.087 | 83.5% | 37.1% | 83.0% | 37.6% | 0.014 |
| Non-binary/third gender | 0.1% | 2.8% | 0.3% | 5.5% | -0.054 | 0.1% | 3.3% | 0.3% | 5.0% | -0.034 |
| **Years in Practice** | | | | | | | | | | |
| 1-5 years in practice | 27.5% | 44.6% | 17.7% | 38.2% | 0.23 | 23.3% | 42.3% | 22.6% | 41.8% | 0.016 |
| 6-10 years in practice | 20.9% | 40.6% | 17.5% | 38.0% | 0.085 | 19.5% | 39.6% | 19.3% | 39.5% | 0.004 |
| 11-15 years in practice | 13.7% | 34.4% | 13.1% | 33.7% | 0.018 | 13.4% | 34.1% | 13.4% | 34.1% | 0.001 |
| 16-20 years in practice | 10.2% | 30.3% | 10.8% | 31.1% | -0.021 | 10.5% | 30.6% | 10.5% | 30.6% | 0 |
| 20+ years in practice | 26.7% | 44.2% | 39.4% | 48.9% | -0.272 | 32.2% | 46.7% | 32.9% | 47.0% | -0.017 |
| Year in practice missing | 1.1% | 10.3% | 1.4% | 11.8% | -0.03 | 1.2% | 10.7% | 1.2% | 11.1% | -0.007 |
| **Practice Setting** | | | | | | | | | | |
| Inpatient Setting | 63.5% | 48.1% | 52.5% | 49.9% | 22.4% | 58.6% | 49.3% | 57.4% | 49.5% | 0.025 |
| Outpatient Setting | 28.3% | 45.0% | 34.7% | 47.6% | -13.7% | 31.3% | 46.4% | 32.1% | 46.7% | -0.018 |
| Setting Missing | 8.2% | 27.4% | 12.9% | 33.5% | -15.4% | 10.1% | 30.2% | 10.5% | 30.7% | -0.013 |

1. **Other Clinical Staff**

| **Unweighted** | | | | | | **Propensity-Weighted** | | | | |
| --- | --- | --- | --- | --- | --- | --- | --- | --- | --- | --- |
|  | **Work Overload Present** | | **Work Overload Not Present** | | **Standardized Difference** | **Work Overload Present** | | **Work Overload Not Present** | | **Standardized Difference** |
|  | **Mean** | **SD** | **Mean** | **SD** |  | **Mean** | **SD** | **Mean** | **SD** |  |
| **COVD Load Quartile** | | | | | | | | | | |
| COVID Quartile 1 | 41.4% | 49.3% | 50.1% | 50.0% | -0.174 | 45.4% | 49.8% | 46.0% | 49.8% | -0.013 |
| COVID Quartile 2 | 3.8% | 19.1% | 4.7% | 21.2% | -0.046 | 4.0% | 19.6% | 4.2% | 20.0% | -0.008 |
| COVID Quartile 3 | 37.3% | 48.4% | 32.0% | 46.6% | 0.111 | 35.3% | 47.8% | 35.0% | 47.7% | 0.006 |
| COVID Quartile 4 | 16.3% | 37.0% | 11.9% | 32.4% | 0.128 | 14.2% | 34.9% | 13.7% | 34.4% | 0.014 |
| COVID Quartile: Missing | 1.1% | 10.5% | 1.3% | 11.2% | -0.014 | 1.1% | 10.3% | 1.1% | 10.3% | 0 |
| **Race/Ethnicity** | | | | | | | | | | |
| Prefer Not to Answer | 10.9% | 31.1% | 8.2% | 27.4% | 0.091 | 9.4% | 29.2% | 9.3% | 29.0% | 0.006 |
| White/Caucasian | 77.3% | 41.9% | 79.6% | 40.3% | -0.056 | 79.2% | 40.6% | 79.2% | 40.6% | 0.001 |
| Hispanic/Latino | 3.4% | 18.0% | 2.2% | 14.6% | 0.071 | 2.8% | 16.5% | 2.7% | 16.2% | 0.005 |
| Black/African American | 3.5% | 18.3% | 2.8% | 16.4% | 0.04 | 3.0% | 17.0% | 3.0% | 17.2% | -0.003 |
| Native American or American Indian | 0.6% | 7.5% | 0.2% | 4.8% | 0.052 | 0.4% | 6.5% | 0.2% | 4.9% | 0.029 |
| Asian/Pacific Islander | 3.6% | 18.6% | 5.7% | 23.1% | -0.099 | 4.2% | 20.0% | 4.5% | 20.7% | -0.015 |
| Other (Please Specify) | 0.9% | 9.4% | 1.4% | 11.7% | -0.046 | 1.0% | 9.9% | 1.1% | 10.5% | -0.01 |
| **Gender** | | | | | | | | | | |
| Prefer not to answer | 9.1% | 28.7% | 5.8% | 23.3% | 0.126 | 7.4% | 26.1% | 7.0% | 25.5% | 0.015 |
| Male | 10.6% | 30.8% | 13.7% | 34.4% | -0.095 | 11.7% | 32.1% | 11.9% | 32.4% | -0.006 |
| Female | 80.2% | 39.9% | 80.5% | 39.6% | -0.008 | 80.9% | 39.3% | 81.1% | 39.1% | -0.007 |
| Non-binary/third gender | 0.1% | 3.3% | 0.0% | 0.0% | 0.047 | 0.1% | 2.8% | 0.0% | 0.0% | 0.032 |
| **Years in Practice** | | | | | | | | | | |
| 1-5 years in practice | 30.6% | 46.1% | 27.0% | 44.4% | 0.078 | 29.0% | 45.4% | 28.7% | 45.2% | 0.005 |
| 6-10 years in practice | 23.0% | 42.1% | 17.9% | 38.3% | 0.125 | 20.4% | 40.3% | 20.4% | 40.3% | 0 |
| 11-15 years in practice | 15.2% | 35.9% | 14.9% | 35.6% | 0.009 | 15.3% | 36.0% | 14.9% | 35.6% | 0.01 |
| 16-20 years in practice | 9.0% | 28.6% | 11.7% | 32.1% | -0.089 | 10.2% | 30.2% | 10.4% | 30.5% | -0.007 |
| 20+ years in practice | 19.8% | 39.9% | 25.8% | 43.7% | -0.141 | 22.6% | 41.8% | 23.0% | 42.1% | -0.009 |
| Year in practice missing | 2.5% | 15.5% | 2.8% | 16.4% | -0.019 | 2.6% | 15.8% | 2.5% | 15.7% | 0.002 |
| **Practice Setting** | | | | | | | | | | |
| Inpatient Setting | 38.4% | 48.6% | 37.2% | 48.3% | 0.025 | 37.6% | 48.4% | 37.5% | 48.4% | 0.002 |
| Outpatient Setting | 49.2% | 50.0% | 46.4% | 49.9% | 0.055 | 48.4% | 50.0% | 48.0% | 50.0% | 0.009 |
| Setting Missing | 12.4% | 33.0% | 16.4% | 37.0% | -0.113 | 13.9% | 34.6% | 14.5% | 35.2% | -0.015 |

1. **Non-Clinical Staff**

| **Unweighted** | | | | | | **Propensity-Weighted** | | | | |
| --- | --- | --- | --- | --- | --- | --- | --- | --- | --- | --- |
|  | **Work Overload Present** | | **Work Overload Not Present** | | **Standardized Difference** | **Work Overload Present** | | **Work Overload Not Present** | | **Standardized Difference** |
|  | **Mean** | **SD** | **Mean** | **SD** |  | **Mean** | **SD** | **Mean** | **SD** |  |
| **COVD Load Quartile** | | | | | | | | | | |
| COVID Quartile 1 | 30.4% | 46.0% | 30.6% | 46.1% | -0.003 | 30.7% | 46.1% | 30.7% | 46.1% | -0.002 |
| COVID Quartile 2 | 4.2% | 20.0% | 4.2% | 20.1% | -0.003 | 4.1% | 19.7% | 4.2% | 20.0% | -0.005 |
| COVID Quartile 3 | 44.8% | 49.7% | 47.2% | 49.9% | -0.047 | 46.0% | 49.8% | 46.3% | 49.9% | -0.005 |
| COVID Quartile 4 | 18.6% | 38.9% | 17.1% | 37.7% | 0.037 | 17.9% | 38.3% | 17.7% | 38.1% | 0.005 |
| COVID Quartile: Missing | 2.0% | 14.1% | 0.9% | 9.5% | 0.093 | 1.4% | 11.9% | 1.2% | 10.8% | 0.021 |
| **Race/Ethnicity** | | | | | | | | | | |
| Prefer Not to Answer | 11.5% | 31.9% | 6.7% | 25.0% | 0.166 | 9.1% | 28.7% | 9.1% | 28.8% | -0.001 |
| White/Caucasian | 77.6% | 41.7% | 83.9% | 36.8% | -0.159 | 81.2% | 39.1% | 81.6% | 38.8% | -0.009 |
| Hispanic/Latino | 2.9% | 16.8% | 2.5% | 15.6% | 0.024 | 2.6% | 15.8% | 2.5% | 15.5% | 0.006 |
| Black/African American | 3.2% | 17.6% | 3.5% | 18.4% | -0.018 | 3.3% | 17.7% | 3.3% | 17.9% | -0.003 |
| Native American or American Indian | 0.4% | 6.2% | 0.5% | 7.1% | -0.017 | 0.3% | 5.9% | 0.4% | 6.6% | -0.014 |
| Asian/Pacific Islander | 3.0% | 17.0% | 2.4% | 15.3% | 0.036 | 2.6% | 16.0% | 2.5% | 15.5% | 0.01 |
| Other (Please Specify) | 1.4% | 12.0% | 0.5% | 7.1% | 0.096 | 0.9% | 9.5% | 0.6% | 7.8% | 0.029 |
| **Gender** | | | | | | | | | | |
| Prefer not to answer | 11.1% | 31.4% | 5.0% | 21.8% | 0.223 | 8.1% | 27.4% | 8.2% | 27.4% | -0.001 |
| Male | 10.3% | 30.4% | 16.8% | 37.4% | -0.19 | 13.3% | 34.0% | 13.5% | 34.2% | -0.004 |
| Female | 78.1% | 41.4% | 78.1% | 41.4% | 0 | 78.2% | 41.3% | 78.2% | 41.3% | 0.001 |
| Non-binary/third gender | 0.5% | 6.9% | 0.1% | 3.2% | 0.071 | 0.3% | 5.4% | 0.2% | 3.9% | 0.026 |
| **Years in Practice** | | | | | | | | | | |
| 1-5 years in practice | 26.4% | 44.1% | 24.5% | 43.0% | 0.042 | 25.5% | 43.6% | 25.5% | 43.6% | 0.002 |
| 6-10 years in practice | 15.7% | 36.3% | 11.3% | 31.7% | 0.127 | 13.6% | 34.3% | 13.3% | 34.0% | 0.008 |
| 11-15 years in practice | 12.2% | 32.7% | 8.6% | 28.1% | 0.116 | 10.5% | 30.6% | 10.3% | 30.4% | 0.005 |
| 16-20 years in practice | 10.7% | 30.9% | 8.9% | 28.5% | 0.061 | 9.9% | 29.8% | 9.9% | 29.9% | -0.001 |
| 20+ years in practice | 21.1% | 40.8% | 25.3% | 43.4% | -0.099 | 22.9% | 42.0% | 23.2% | 42.2% | -0.006 |
| Year in practice missing | 14.0% | 34.7% | 21.3% | 41.0% | -0.193 | 17.6% | 38.1% | 17.8% | 38.3% | -0.006 |
| **Practice Setting** | | | | | | | | | | |
| Inpatient Setting | 34.3% | 47.5% | 28.1% | 44.9% | 0.135 | 31.2% | 46.3% | 31.1% | 46.3% | 0.003 |
| Outpatient Setting | 30.9% | 46.2% | 28.0% | 44.9% | 0.065 | 29.2% | 45.5% | 29.2% | 45.5% | 0 |
| Setting Missing | 34.8% | 47.6% | 44.0% | 49.6% | -0.188 | 39.6% | 48.9% | 39.8% | 48.9% | -0.004 |
